# Anti‐SARS‐CoV‐2 IgG antibody levels among Thai healthcare providers receiving homologous and heterologous COVID‐19 vaccination regimens

**DOI:** 10.1111/irv.12975

**Published:** 2022-02-24

**Authors:** Wanitchaya Kittikraisak, Taweewun Hunsawong, Somsak Punjasamanvong, Thanapat Wongrapee, Patama Suttha, Phunlerd Piyaraj, Chaniya Leepiyasakulchai, Chuleeekorn Tanathitikorn, Pornsak Yoocharoen, Anthony R. Jones, Duangrat Mongkolsirichaikul, Matthew Westercamp, Eduardo Azziz‐Baumgartner, Joshua A. Mott, Suthat Chottanapund

**Affiliations:** ^1^ Influenza Program Thai Ministry of Public Health – U.S. Centers for Disease Control and Prevention Nonthaburi Thailand; ^2^ Virology Department Armed Forces Research Institute of Medical Sciences Bangkok Thailand; ^3^ Internal Medicine Department Rayong Hospital Rayong Thailand; ^4^ Internal Medicine Department Phaholpolpayuhasena Hospital Kanchanaburi Thailand; ^5^ Internal Medicine Department Bamrasnaradura Infectious Diseases Institute Nonthaburi Thailand; ^6^ Parasitology Department Phramongkutklao College of Medicine Bangkok Thailand; ^7^ Faculty of Medical Technology Mahidol University Nakhon Pathom Thailand; ^8^ Department of Disease Control Ministry of Public Health Nonthaburi Thailand; ^9^ Division of Healthcare Quality Promotion U.S. Centers for Disease Control and Prevention Atlanta Georgia USA; ^10^ Influenza Division U.S. Centers for Disease Control and Prevention Atlanta Georgia USA

**Keywords:** COVID‐19, healthcare provider, IgG antibody, SARS‐CoV‐2, Thailand, vaccination

## Abstract

**Background:**

We examined SARS‐CoV‐2 anti‐spike 1 IgG antibody levels following COVID‐19 vaccination (AstraZeneca [AZ], Sinovac [SV], Pfizer‐BioNTech [PZ]) among Thai healthcare providers.

**Methods:**

Blood specimens were tested using enzyme‐linked immunosorbent assay. We analyzed seven vaccination regimens: (1) one dose of AZ or SV, (2) two doses of homologous (2AZ, 2SV) or heterologous (1AZ + 1PZ) vaccines, and (3) three doses of heterologous vaccines (2SV + 1AZ, 2SV + 1PZ). Differences in antibody levels were assessed using Kruskal–Wallis statistic, Mann–Whitney test, or Wilcoxon matched‐pairs signed‐rank test. Antibody kinetics were predicted using fractional polynomial regression.

**Results:**

The 563 participants had median age of 39 years; 92% were female; 74% reported no underlying medical condition. Antibody levels peaked at 22–23 days in both 1AZ and 2SV vaccinees and dropped below assay's cutoff for positive (35.2 binding antibody units/ml [BAU/ml]) in 55 days among 1AZ vaccinees compared with 117 days among 2SV vaccinees. 1AZ + 1PZ vaccination regimen was highly immunogenic (median 2279 BAU/ml) 1–4 weeks post vaccination. 2SV + 1PZ vaccinees had significantly higher antibody levels than 2SV + 1AZ vaccinees 4 weeks post vaccination (3423 vs. 2105 BAU/ml; *p*‐value < 0.01), and during weeks 5–8 (3656 vs. 1072 BAU/ml; *p*‐value < 0.01). Antibodies peaked at 12–15 days in both 2SV + 1PZ and 2SV + 1AZ vaccinees, but those of 2SV + 1AZ declined more rapidly and dropped below assay's cutoff in 228 days while those of 2SV + 1PZ remained detectable.

**Conclusions:**

1AZ + 1PZ, 2SV + 1AZ, and 2SV + 1PZ vaccinees had substantial IgG levels, suggesting that these individuals likely mounted sufficient anti‐S1 IgG antibodies for possible protection against SARS‐CoV‐2 infection.

## 1 INTRODUCTION

In March 2020, the World Health Organization (WHO) characterized the novel coronavirus disease 2019 (COVID‐19) outbreak, caused by severe acute respiratory syndrome coronavirus 2 (SARS‐CoV‐2), as a global pandemic.^1^ As of January 2022, 388 million people worldwide have been cumulatively reported to be infected with SARS‐CoV‐2 and 5.7 million deaths registered.^2^ Individuals of any age with underlying medical conditions, older adults (≥65 years), pregnant women, and smokers are at higher risk of severe illness.^3^, ^4^, ^5^, ^6^ Those in the healthcare sector are disproportionately affected by COVID‐19.^7^, ^8^ Despite sustained epidemics in many countries, a systematic review of studies including 6.3 million individuals from 60 countries indicated that by mid‐2021 the majority of the world's human population was still susceptible to SARS‐CoV‐2 infection, with a pooled seroprevalence of approximately 10%.^9^

Safe and effective COVID‐19 vaccines are essential to reduce COVID‐19 morbidity and mortality. COVID‐19 vaccines are now available.^10^ As of December 2021, one COVID‐19 vaccine (BNT162b2, Pfizer‐BioNTech [PZ]) has received full approval from the U.S. Food and Drug Administration for use in individuals aged ≥16 years,^11^ whereas others (e.g., ChAdOx1 nCoV‐19, also known as AstraZeneca [AZ], CoronaVac, also known as Sinovac [SV]) are authorized for emergency use only by the WHO. COVID‐19 vaccines elicit detectable antibodies in early stages, but the antibody levels wane over time.^12^, ^13^, ^14^, ^15^, ^16^ For example, a study among 3808 PZ vaccinees reported that humoral response decreased 6 months after completion of the primary series.^15^ Further, infections with different SARS‐CoV‐2 variants among persons immunized with all COVID‐19 vaccine products have been reported,^17^, ^18^, ^19^, ^20^ prompting countries to adjust vaccination regimens based on resources and vaccine availability.

Thailand started COVID‐19 vaccination of healthcare providers (HCPs) and community health volunteers on February 28, 2021.^21^ By September 2021, 800,000 HCPs and community health volunteers completed the primary series of the vaccines.^21^ The majority of these individuals received two doses of an inactivated vaccine (SV), whereas others received two doses of a vector‐based vaccine (AZ). Because of increased identification of the SARS‐CoV‐2 Delta variant in Thailand in April 2021, the Ministry of Public Health (MOPH) recommended that those receiving the primary series of SV, and who cared for COVID‐19 patients, be given a booster vaccine dose with different product (i.e., heterologous vaccination) ≥4 weeks after the second dose of SV.^22^ HCPs were given the choice of which vaccine product (vector‐based or mRNA‐based) to use for their booster COVID‐19 dose. The currently available data suggest that the primary series of prime‐boost heterologous vaccination combination of AZ/PZ and rAd26/rAd5 (Sputnik) induce a strong and broad immune response in healthy individuals.^23^, ^24^, ^25^, ^26^, ^27^, ^28^, ^29^ Immunogenicity data on other heterologous vaccination regimens (i.e., 2SV with AZ or PZ booster), however, are limited.

To address the knowledge gap of immunogenicity following vaccination with 2SV with AZ or PZ booster, this report details the kinetics and anti‐spike 1 protein (S1) IgG antibody levels elicited by COVID‐19 vaccination, as measured by enzyme‐linked immunosorbent assay (ELISA). This study is part of an ongoing prospective cohort that follows HCPs for 2 years for SARS‐CoV‐2 immune response, illness incidence, illness cost, and exposure risk.

## 2 MATERIAL AND METHODS

### 2.1 Study setting

In January 2021, we established a cohort of 600 HCPs based on convenience sampling in four Thai hospitals: Bamrasnaradura Infectious Diseases Institute (BIDI, total 819 HCPs); Phaholpolpayuhasena Hospital (PH, total 1400 HCPs); Phramongkutklao Hospital (PMK, total 1428 HCPs); and Rayong Hospital (RY, total 1600 HCPs). We defined HCPs as individuals providing direct healthcare services (e.g., vital sign measurement, bathing or examining patients, taking specimens from patients) in a healthcare setting. Eligible HCPs were those who aged ≥18 years, worked ≥30 h/week, and cared for ≥1 patient per day. Those who were employed <1 year or acutely ill with COVID‐19 at enrollment were ineligible. Study staff contacted the participants through a messaging application weekly to inquire about symptoms in the past 7 days (cough, runny nose/congestion, sore throat, difficult breathing, or muscle pain). Those with any symptom were offered real‐time reverse transcription polymerase chain reaction (rRT‐PCR) testing for SARS‐CoV‐2. Additionally, participants had access to testing at their hospitals when SARS‐CoV‐2 exposure was suspected.

### 2.2 Verification of COVID‐19 vaccination

Participants shared a copy of their government‐issued documents with study staff to verify their vaccination information.

### 2.3 Blood collection and processing

Blood specimens were collected from all participants at enrollment, and every 3 months thereafter according to the study's schedule (regardless of participants' vaccination timelines). As vaccination occurred in phases, this resulted in different time intervals from vaccination to blood collection among participants (i.e., those receiving the vaccines early having longer intervals from vaccination to blood collection than those receiving the vaccines late). Heparinized blood specimens were collected and transported to Mahidol University's laboratory. The separated plasma was stored at −20°C until transported to the Armed Forces Research Institute of Medical Sciences' laboratory.

### 2.4 Laboratory testing

The thawed plasma was screened for the presence of anti‐S1 IgG antibodies using the Thai and U.S. Food and Drug Administration Emergency Use Authorized EUROIMMUN kit (Lübeck, Germany; catalog number: EI2606‐9601‐10G). To test, diluted plasma was incubated in reaction wells each coated with recombinant SARS‐CoV‐2 spike protein. Specific IgG antibodies that bounded to the to the spike protein antigen were detected using an enzyme‐conjugated colorimetric technique. The quantitative results were calculated as a ratio of the extinction of color of the control or tested specimen over the extinction of calibrators. The anti‐S1 IgG antibody levels, reported as binding antibody units/ml (BAU/ml), were calculated using a standard curve generated from 6‐point calibrators. Per the manufacturer, antibody levels of ≥35.2 BAU/ml were considered positive.

### 2.5 Data analysis

For analyses, SARS‐CoV‐2 rRT‐PCR‐confirmed cases during follow‐up or those testing positive for anti‐S1 IgG antibodies at enrollment, HCPs who self‐reported immunocompromised status or history of cancer, and those who did not provide blood specimens at months three and six visits were excluded (*n* = 37). The 563 remaining participants were grouped based on number of COVID‐19 vaccine doses received prior to each blood collection and vaccine product. We analyzed seven vaccination regimens: (1) one dose of AZ or SV (1AZ, 1SV); (2) two doses of homologous vaccines (two doses of AZ [2AZ], two doses of SV [2SV]); (3) two doses of heterologous vaccines (AZ and PZ [1AZ + 1PZ]); and (4) three doses of heterologous vaccines (two doses of SV with an AZ or PZ booster [2SV + 1AZ or 2SV + 1PZ]). Each participant contributed up to three blood specimens for analyses.

By month six visit, two participants did not receive COVID‐19 vaccines. Of 561 who did, 533 (95.0%) transitioned from one vaccination regimen to another (e.g., 1SV at month three visit to 2SV at month six visit). When transition did not occur, only data points from the latest blood collection within the same vaccination regimen were used to create an analytic dataset with independent observations. Days from the last dose were calculated as duration between last vaccination and blood collection. We assessed differences in antibody levels between time periods using the Kruskal‐Wallis (>2 groups), or the Mann–Whitney (two groups) test, as appropriate. When comparing the same individuals transitioning from 2SV to 2SV + 1AZ or 2SV + 1PZ regimen, the Wilcoxon matched‐pairs signed‐rank test was used. We used a level of 264 BAU/ml as a threshold for 80% protection against SARS‐CoV‐2 symptomatic infection with the Alpha variant.^30^ We predicted the median antibody levels from estimation of a fractional polynomial of days from the last dose and plotted the resulting curve along with the 95% confidence interval of the median.^31^ Data analysis was performed using Stata version 16 (Stata Corp., USA) and GraphPad version 9.2 (GraphPad Software, Inc., USA); *p*‐values of <0.05 indicated statistical significance.

### 2.6 Ethics approval and informed consent

This study was approved by the Institutional Review Boards (IRBs) of BIDI; PH; PMK; RY; Department of Disease Control of the Thai MOPH (Thailand); and Walter Reed Army Institute of Research (USA). The IRBs of the U.S. Centers for Disease Control and Prevention (USA) and Mahidol University (Thailand) relied on the determinations of PMK's and MOPH's IRBs, respectively. All participants provided written informed consent.

## 3 RESULTS

### 3.1 Baseline characteristics of study participants

**FIGURE 1:**
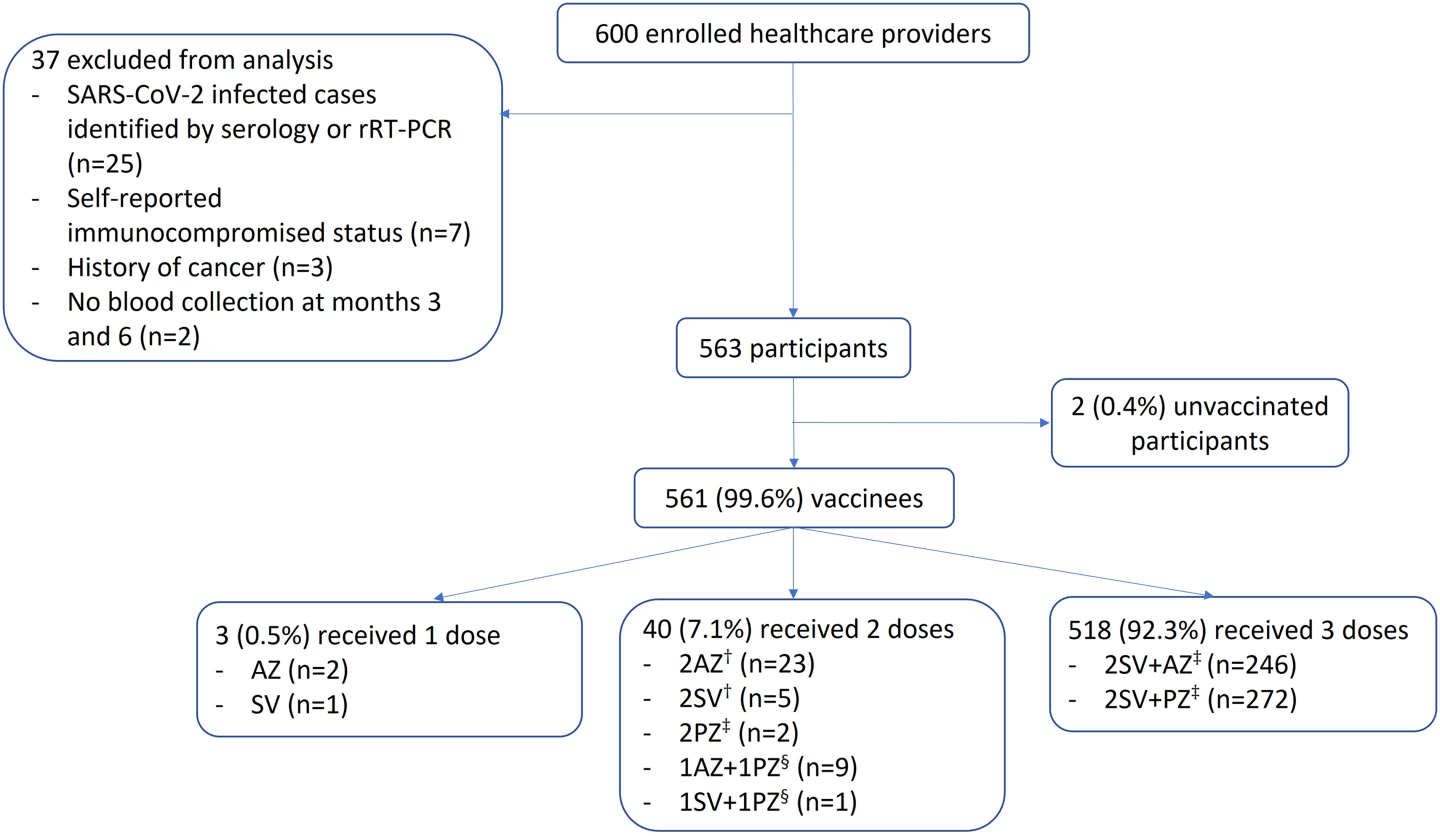
Enrollment flow among study participants who enrolled into a healthcare provider cohort, Bangkok, Thailand (2021). rRT‐PCR, real‐time reverse transcription polymerase chain reaction; AZ, AstraZeneca; SV, Sinovac; PZ, Pfizer‐BioNTech; n, number. Participants contributed data to multiple vaccination strategies. Vaccine strategies shown here reflect doses received by the time of month six blood collection. ^†^Thai Ministry of Public Health's guideline during the commencement of COVID‐19 vaccination campaign in Thailand in late February 2021: (1) two doses of Sinovac (0.5 ml) given intramuscularly with 2–4 weeks interval between the first and second doses; (2) two doses of AstraZeneca (0.5 ml) given intramuscularly with 8–12 weeks interval between the first and second doses. ^‡^Thai Ministry of Public Health's guideline issued in June 2021: (1) two doses of Pfizer‐BioNTech (0.3 ml) given intramuscularly with 3 weeks interval between the first and second doses; (2) one dose of AstraZeneca or Pfizer‐BioNTech (0.3 ml) given intramuscularly 4 weeks after completion of the primary series of Sinovac. ^§^Not in Thai Ministry of Public Health's guideline as of June 2021

From January 18, 2021 to March 5, 2021, we enrolled 600 HCPs. Of the 563 participants analyzed (Figure 1), the median enrollment age was 39 years (interquartile range 29–48; Table 1); four participants were >60 years old (range 61–63). The majority (519; 92.2%) were female. Three hundred and seventy (65.7%) were nurses, 109 (19.4%) nurse aids, 17 (3.0%) physicians, 11 (1.9%) laboratory technicians, and 56 (9.9%) had other professions involving patient care. Four hundred and sixteen (73.9%) participants reported no underlying medical condition, 144 (25.6%) had ≥1 underlying condition, and 3 (0.5%) were unsure of underlying medical conditions. Metabolic diseases (including diabetes) were the most common (79; 54.9%; Table S1).

**TABLE 1:** Characteristics at enrollment of 563 study participants who enrolled into a healthcare provider cohort, Bangkok, Thailand (2021)

Characteristics	Number of participants (%)
Age (years)	39 (29–48)^†^
Sex	
Male	44 (7.8)
Female	519 (92.2)
Profession	
Nurse	370 (65.7)
Nurse aid	109 (19.4)
Physician	17 (3.0)
Laboratory technician	11 (1.9)
Others^‡^	56 (9.9)
Number of pre‐existing medical conditions	
Not sure	3 (0.5)
0	416 (73.9)
1	111 (19.7)
2–3	33 (5.9)

^†^ Median (interquartile).

^‡^ Assistant to dentist, triage staff, technician for coronary artery angiography, technician at sport medicine center, and paramedic.

### 3.2 COVID‐19 vaccination

No participants had received any COVID‐19 vaccination at the time of enrollment; however, all but two (99.6%) were vaccinated by their month six visit. Among these, 3 (0.5%) received one dose, 40 (7.1%) received two doses, and 518 (92.3%) received three doses. Thirty of 40 (75.0%) HCPs with two vaccine doses received homologous vaccination, whereas 10 (25.0%) received heterologous vaccination (Figure 1). The primary series of AZ was administered with a median of 91 (interquartile range [IQR], 83–91; range 60–93) days between doses (recommended interval was 56–84 days), whereas those with SV and PZ were a median of 21 (IQR, 21–28; range 21–30) and 24 (IQR, 22–27; range 22–27) days between doses, respectively (recommended intervals were 14–28 days for SV and 21 days for PZ; Table 2). There was no notable fraction of participants having long delays between vaccine doses. Among 2SV vaccinees who received the third vaccine dose, 246 (47.5%) and 272 (52.5%) received AZ and PZ, respectively. Those receiving the AZ booster dose were significantly older than those receiving the PZ booster dose (*p*‐value < 0.01).

**TABLE 2:** COVID‐19 vaccination and time interval between doses among 563 study participants who enrolled into a healthcare provider cohort, Bangkok, Thailand (2021)

Vaccination strategy^†^	Number of participants	Age at enrollment in years (IQR)	Number of days reported as median and IQR	
			Between 1^st^ and 2^nd^ doses	Between 2^nd^ and 3^rd^ doses
Unvaccinated	2	51.5 (49–54)	n/a	n/a
AZ	2	40.5 (40–41)	n/a	n/a
SV	1	35 (n/a)	n/a	n/a
2AZ^‡^	23	46 (31–57)	91 (83–91)	n/a
2SV^‡^	5	30 (25–32)	27 (26–28)	n/a
2PZ^§^	2	28.5 (28–29)	24 (22–27)	n/a
1AZ + 1PZ^¶^	9	43 (37–46)	64 (55–65)	n/a
1SV + 1PZ^¶^	1	42 (n/a)	133 (n/a)	n/a
2SV + 1AZ^§^	246	41 (32–50)	21.5 (21–28)	86 (68–91)
2SV + 1PZ^§^	272	35 (28–45)	21 (21–28)	102.5 (89–111)

Abbreviations: AZ, AstraZeneca; SV, Sinovac; PZ, Pfizer‐BioNTech; IQR, interquartile; n/a, not applicable.

^†^ Participants contributed data to multiple vaccination strategies. Vaccine strategies shown here reflect doses received by the time of month six blood collection.

^‡^ Thai Ministry of Public Health's guideline during the commencement of COVID‐19 vaccination campaign in Thailand in late February 2021: (1) two doses of Sinovac (0.5 ml) given intramuscularly with 2–4 weeks interval between the first and second doses; (2) two doses of AstraZeneca (0.5 ml) given intramuscularly with 8–12 weeks interval between the first and second doses.

^§^ Thai Ministry of Public Health's guideline issued in June 2021: (1) two doses of Pfizer‐BioNTech (0.3 ml) given intramuscularly with 3 weeks interval between the first and second doses; (2) one dose of AstraZeneca or Pfizer‐BioNTech (0.3 ml) given intramuscularly 4 weeks after completion of the primary series of Sinovac.

^¶^ Not in Thai Ministry of Public Health's guideline as of June 2021.

### 3.3 Anti‐S1 IgG antibody response after the first dose

**FIGURE 2:**
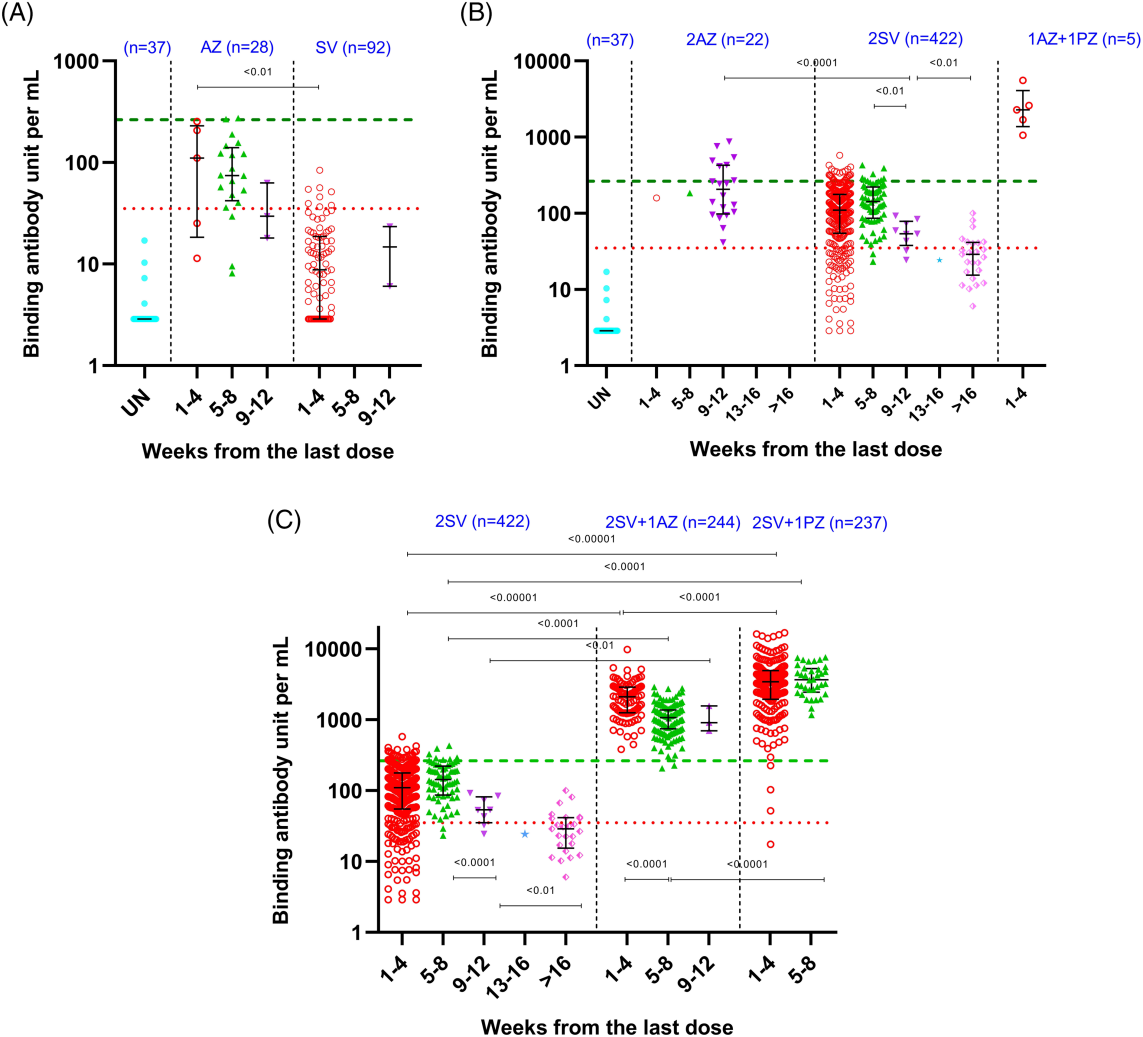
Anti‐S1 IgG antibody response among study participants who enrolled into a healthcare provider cohort, Bangkok, Thailand (2021). (A) After one dose of AstraZeneca or Sinovac. (B) After two doses of homologous and heterologous vaccinations. (C) After two doses of Sinovac with and without booster dose (AstraZeneca or Pfizer‐BioNTech). UN, unvaccinated at the time of blood collection; AZ, AstraZeneca; SV, Sinovac; PZ, Pfizer‐BioNTech. Red, green, purple, blue, and pink symbols on figures are data points. N indicates number of data points. Red dotted line indicates assay's cutoff for positive (i.e., 35.2 binding antibody units/ml [BAU/ml]). Green dashed line indicates level (264 BAU/ml) suggested for 80% protection against symptomatic infection with SARS‐CoV‐2 Alpha variant.^30^ Black line and error bars indicate median and interquartile range

During the first 12 weeks following one dose of AZ and SV, 21 (75.0%) of 28 and 8 (8.7%) of 92 vaccinees, respectively, had anti‐S1 IgG antibody levels above the assay's cutoff level for a positive result (Figure 2A). Neither of unvaccinated participants had detectable anti‐S1 IgG antibodies. Two (7.1%) of 28 1AZ vaccinees compared with none (0%) of 92 1SV vaccinees had antibody levels above the threshold suggested for 80% protection against SARS‐CoV‐2 symptomatic infection. Vaccinees with a single dose of AZ or SV had no statistically significant change in the median anti‐S1 IgG antibody levels throughout the 12 weeks post vaccination (*p*‐value = 0.26 and *p*‐value = 0.59, respectively), although the data points among 1SV vaccinees were limited after 4 weeks. The anti‐S1 IgG antibody levels among 1AZ vaccinees (median 111 BAU/ml, IQR 25–207) were significantly higher than those of 1SV vaccinees (median 9 BAU/ml, IQR 3–19; *p*‐value < 0.01) during the first 4 weeks post vaccination (Figure 2A).

### 3.4 Anti‐S1 IgG antibody response after two doses of homologous and heterologous vaccinations

During the first 12 weeks following completion of homologous 2AZ vaccination, all vaccinees (22/22; 100.0%) had detectable anti‐S1 IgG antibodies, in comparison to 334 (84.4%) of 397 homologous 2SV vaccinees (Figure 2B). Eight (32.0%) of 25 homologous 2SV vaccinees also had detectable anti‐S1 IgG antibody levels at weeks >12 (data points for >12 weeks were limited for the 2AZ vaccinees and not analyzed). Seven (31.8%) of 22 homologous 2AZ vaccinees and 33 (8.3%) of 397 homologous 2SV vaccinees had antibody levels above the threshold for 80% protection during the first 12 weeks. The available data showed that these 22 2AZ vaccinees had a median anti‐IgG antibody level of 207 BAU/ml (IQR 100–423) during weeks 9–12. For homologous 2SV vaccination, the overall anti‐S1 IgG antibody levels declined significantly over time (*p*‐value < 0.01), especially from weeks 5–8 (median 143 BAU/ml, IQR 86–220) to weeks 9–12 (median 54 BAU/ml, IQR 43–73; *p*‐value < 0.01), and from weeks 9–12 to weeks >16 (median 29 BAU/ml, IQR 17–41; *p*‐value < 0.01).

Among five participants for whom data during the first 4 weeks post vaccination were available, heterologous 1AZ + 1PZ vaccination regimen was highly immunogenic (median 2279 BAU/ml, IQR 1690–2609; Figure 2B). All of these vaccinees had anti‐S1 IgG antibody levels exceeding the threshold for 80% protection.

For weeks 9–12 (when direct comparisons between vaccination regimens were possible), the anti‐S1 IgG antibody levels among homologous 2AZ vaccination (median 207 BAU/ml, IQR 100–423) were significantly higher than those of homologous 2SV vaccination (median 54 BAU/ml, IQR 43–73; *p*‐value < 0.01). Similarly, the anti‐S1 IgG antibody levels among heterologous 1AZ + 1PZ vaccinees (median 2279 BAU/ml, IQR 1690–2609) were significantly higher than those of homologous 2SV vaccinees (median 110 BAU/ml, IQR 54–118) during weeks 1–4 post vaccination (*p*‐value < 0.01).

### 3.5 Anti‐S1 IgG antibody response after the third dose

During the first 12 weeks post vaccination with the third vaccine dose, all 244 2SV + 1AZ vaccinees had detectable anti‐S1 IgG antibody levels; these antibody levels exceeded the threshold for 80% protection in 242 (99.2%) of 244 vaccinees (Figure 2C). Antibody levels in heterologous 2SV + 1AZ vaccinees decreased significantly from weeks 1–4 (median 2105 BAU/ml, IQR 1253–2881) to weeks 5–8 (median 1072 BAU/ml, IQR 750–1375; *p*‐value < 0.01), although there was no difference in antibody levels between weeks 5–8 and 9–12 (median 1166 BAU/ml, IQR 1802–1497; *p*‐value = 0.78).

During the first 8 weeks post vaccination with the third vaccine dose, 236 (99.6%) of 237 2SV + 1PZ vaccinees had detectable anti‐S1 IgG antibody levels (Figure 2C). Among these 237 vaccinees, 233 (98.3%) had antibody levels exceeding the threshold for 80% protection. There was no significant change in antibody levels among 2SV + 1PZ vaccinees between weeks 1–4 (median 3423 BAU/ml, IQR 1973–4943) and weeks 5–8 (median 3656 BAU/ml, IQR 2516–5220; *p*‐value = 0.18).

With a median anti‐S1 IgG antibody level of 3423 BAU/ml (IQR 1973–4943), the PZ booster elicited higher antibody levels than the AZ booster (median 2105 BAU/ml, IQR 1253–2881) during first 4 weeks post vaccination (*p*‐value < 0.01), and during weeks 5–8 (median 3656 BAU/ml, IQR 2516–5220 vs. median 1072 BAU/ml, IQR 750–1375; *p*‐value < 0.01).

### 3.6 Anti‐S1 IgG antibody kinetics

**FIGURE 3:**
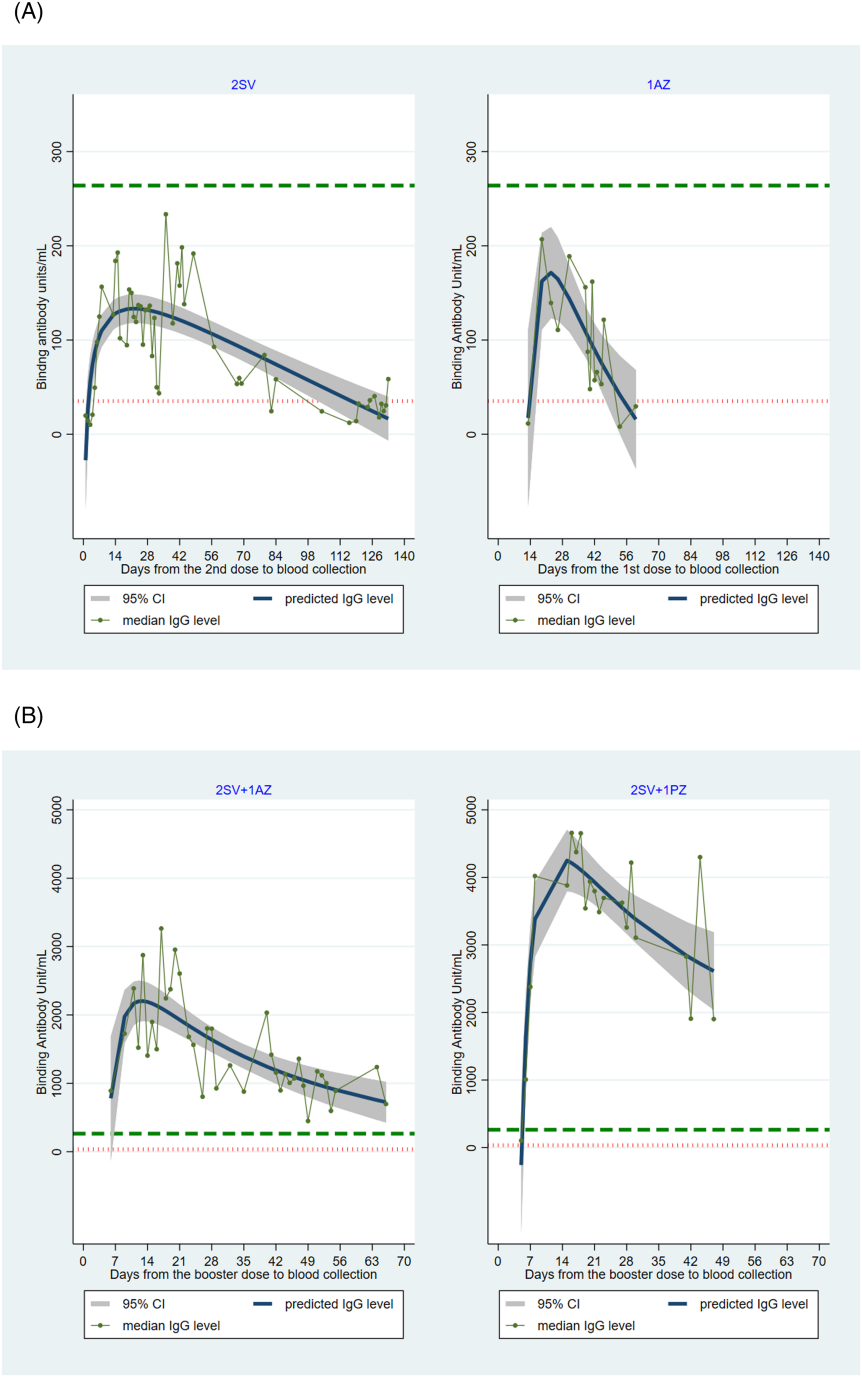
Anti‐S1 IgG antibody kinetics by days since the last dose of COVID‐19 vaccination. (A) Vaccinees who received two doses of Sinovac compared with those who received one dose of AstraZeneca. (B) Vaccinees who received AstraZeneca or Pfizer‐BioNTech booster dose following a complete series of Sinovac. SV, Sinovac; AZ, AstraZeneca; PZ, Pfizer‐BioNTech. Red dotted line indicates assay's cutoff for positive (i.e., 35.2 binding antibody units/ml [BAU/ml]). Green dashed line indicates level (264 BAU/ml) suggested for 80% protection against symptomatic infection with SARS‐CoV‐2 Alpha variant.^30^ The median anti‐Spike 1 IgG antibody level was predicted from an estimation of fractional polynomial regression of days from the last dose; the resulting curve was plotted along with the 95% confidence interval of the median. Equations used to estimate the predicted anti‐S1 IgG antibody levels in binding antibody units/ml are as follows: (1) $\mathrm{two}\;\text{doses of Sinovac}=17+\left(106\times \ln\left(\text{days}\right)\right)-\left(45\times \sqrt{\text{days}}\right)$; (2) $\mathrm{one}\;\text{dose of AstraZeneca}=-495-\left(\frac{110002}{{\text{days}}^{2}}\right)+\left(\frac{4194}{\sqrt{\text{days}}}\right)$; (3) $\text{AstraZeneca after completion of}\;\mathrm{two}\;\text{dose of Sinovac}=-877-\left(\frac{141597}{{\text{days}}^{2}}\right)+\left(\frac{13822}{\surd \text{days}}\right)$; and (4) $\text{Pfizer after completion of}\;\mathrm{two}\;\text{dose of Sinovac}=-655-\left(\frac{106227}{\text{days}}\right)+(\frac{67281}{\text{days}\;}x\;\ln\left(\text{days}\right)$)

Figure 3A shows the kinetics of anti‐S1 IgG antibody levels by day since the last dose among 2SV and 1AZ vaccinees. In both groups, anti‐S1 IgG antibody levels peaked at days 22–23 and then declined as time progressed. However, the median anti‐S1 IgG antibody level among 1AZ vaccinees appeared to decline more rapidly than that of 2SV vaccinees and was predicted to drop below the assay's cutoff level for positive in 55 days compared with 117 days among 2SV vaccinees (Figure 3A). The number of 2AZ vaccinees was too small for the kinetics assessment.

The antibody kinetics among 2SV + 1AZ vaccinees differed from those of 2SV + 1PZ vaccinees (Figure 3B). Although the anti‐S1 IgG antibody levels peaked around 12–15 days in both groups, the median antibody level among 2SV + 1AZ vaccinees declined during the observed period and was predicted to drop below level suggested for 80% protection in 145 days and below the assay's cutoff level for positive in 228 days. A much stronger response was observed among 2SV + 1PZ vaccinees with median antibody level remaining above the threshold for positive throughout the observed period. At 228 days when the median antibody level among 2SV + 1AZ dropped below the assay's cutoff for positive, that of the 2SV + 1PZ was predicted to remain at 471 BAU/ml (Figure 3B).

## 4 DISCUSSION

In this study of 563 individuals, we demonstrated that AZ and SV elicited humoral immune response among a generally healthy cohort of participants. Serial vaccination increased the proportion of seropositive persons. In addition, the homologous 2AZ and heterologous 1AZ + 1PZ vaccination regimens elicited higher antibody levels than that of the 2SV. During the period that this cohort had been followed to the time this report was written, those receiving AZ or PZ as a third dose following the completion of a primary series of SV mounted substantial anti‐S1 IgG antibody levels that exceeded the threshold suggested for 80% protection. However, antibody levels of the 2SV + 1AZ vaccinees declined and were estimated to drop below the assay's cutoff level for positive in 228 days (about 7.5 months) after the third dose whereas those of 2SV + 1PZ vaccinees remained detectable.

The detection of anti‐S1 IgG antibodies reflects an immune response to vaccination and some clinical protection from SARS‐CoV‐2 infection. However, knowledge about the level of antibodies rendering vaccine effectiveness/efficacy is still limited, and antibody levels do not reflect cellular immune response which may also influence longer‐term immunity. Higher levels of immune response are believed to be associated with a reduced risk of infection and illness attenuation.^30^, ^32^ A recent study by Feng et al. suggested that a vaccine efficacy of 80% against symptomatic infection with majority SARS‐CoV‐2 Alpha variant was achieved with anti‐S1 IgG antibody levels of ≥264 BAU/ml whereas levels of ≥29 BAU/ml conferred 50% protection.^30^ We found that during the study period, participants had anti‐S1 IgG antibody levels above the threshold for 80% protection after receiving homologous 2AZ (32%) and 2SV (8%), and heterologous 1AZ + 1PZ (100%) vaccination regimens. The effect was more pronounced when the third vaccine dose was given, suggesting that these individuals likely mounted sufficient anti‐S1 IgG antibodies for possible protection against SARS‐CoV‐2 infection. Generally, the anti‐S1 IgG antibodies among 2SV + 1AZ vaccinees were in the same magnitude as the levels, measured 14 days post vaccination, among healthy HCPs completing two doses of PZ in other study.^33^ Likewise, the anti‐S1 IgG antibody levels among 2SV + 1PZ were similar to the antibody levels among healthy adults who were primed with AZ and boosted with PZ.^34^

Our data corroborate other studies that reported SV‐, AZ, and PZ‐elicited antibodies wane over time although the rate of decline is unclear, and the impact on protection against infection and on illness attenuation is undefined.^12^, ^13^, ^14^, ^15^, ^16^, ^35^ In this study, a significant reduction of antibody levels was observed among participants who completed the primary series of SV and those receiving 2SV + 1AZ, in a relatively short period (i.e., during the observed time). Nonetheless, from an immunological standpoint, the waning of these antibodies is expected. Similar to any vaccination, other components of adaptive immunity such as memory B‐ and T‐lymphocytes may also play a role in the elimination of invading pathogens following vaccination. This highlights the importance of studying cellular immune response to better understand the role of these memory cells in longer‐term protection against infection and severity of illness.^36^

We used the threshold of 264 BAU/ml as a proxy of anti‐S1 IgG antibody level conferring 80% protection against symptomatic infection to the SARS‐CoV‐2 Alpha variant.^30^ It is not known if this suggested threshold will change as different variants rising to predominance are further studied. This poses challenges to the control of the pandemic as some variants have increased ability to escape the immune response and/or are more contagious.^37^, ^38^, ^39^, ^40^ Viral neutralization tests also showed that the elicited antibodies had different efficiency in neutralizing SARS‐CoV‐2 lineages.^41^ In Thailand, detection of other SARS‐CoV‐2 variants such as the Delta and the Omicron has been reported.^42^ There is a public health need to quickly re‐assess the proportions of vaccinated individuals protected when levels for correlate of protection against additional SARS‐CoV‐2 variants of concern are firmly established. This is of particular importance as we observed the emergence of cases infected with the Delta variant among cohort participants in July 2021. This multi‐site platform is thus ideal to understand the immunologic response among vaccinated persons to novel SARS‐CoV‐2 strains.

The design and timing of this study were strengths. Our study was well timed to the epidemic in Thailand as all participants were enrolled prior to when COVID‐19 vaccines became available, giving us the opportunity to include all relevant time points (baseline prior to vaccination, followed by 3‐month follow‐up visits). The study also provided an opportunity to examine various COVID‐19 vaccination regimens. Vaccine acceptance rate was high (99%), and we verified vaccination information in all participants. The comprehensive follow‐up, which included active weekly contact and rRT‐PCR testing, allowed for timely identification of SARS‐CoV‐2 infected cases. Participants with prior or active SARS‐CoV‐2 infection were excluded from analyses to provide an unconfounded overview of humoral immune response to COVID‐19 vaccination. We reported findings using STrengthening the Reporting of OBservational studies in Epidemiology (STROBE) guidelines^43^ and presented antibody results in standardized units, facilitating comparison across studies which may use different test kits.^44^

This study also had some limitations. Females comprised the majority of our HCP population. Our results were limited to approximately the first 4 months following vaccination. Continued follow‐up of cohort participants is warranted to study the longevity of vaccine‐elicited immune response and the impact of SARS‐CoV‐2 variants on infection in vaccinated persons over time. The sample sizes and follow‐up time were limited in some vaccination regimens. Although no adverse events were reported to study staff, we did not systematically assess reactogenicity following vaccination. In addition, the 2SV + 1AZ vaccinees were significantly older than the 2SV + 1PZ counterparts so observed differences may be due to immunosenescent or comorbidities rather than vaccine products. Further, the assay used to detect binding antibodies in this study was based on the conserved S1 domain of the spike protein of SARS‐CoV‐2 Wuhan strain, whereas mutations in this particular region were well documented in most SARS‐CoV‐2 variants of concern identified to date.^45^ Lastly, relationship between binding and neutralizing antibodies in protective antiviral immunity following COVID‐19 vaccination is currently not well understood. Results on antibody function and the potential role of memory B‐ and T‐lymphocyte response using polymorphonuclear cells harvested from these vaccinees and those naturally infected with SARS‐CoV‐2 are still pending.

## 5 CONCLUSIONS

Our study contributed to the limited evidence of the impact of diverse COVID‐19 vaccination regimens. Homologous 2AZ and 2SV vaccination regimens elicited good humoral immune response following the completion of the primary series, but an especially potent antibody response was observed among heterologous 1AZ + 1PZ vaccinees. Substantial increases in anti‐S1 IgG antibody levels after a booster dose of AZ or PZ were observed among those who completed the primary series of SV, although there were meaningful differences in product performance. These findings suggested that some heterologous vaccination regimens may generate vigorous humoral immune response in generally healthy adults.

## AUTHOR CONTRIBUTIONS

**Wanitchaya Kittikraisak:** Conceptualization; formal analysis; methodology; project administration; resources; supervision; validation. **Taweewun Hunsawong:** Investigation; methodology; resources; supervision. **Somsak Punjasamanvong:** Project administration; resources; supervision. **Thanapat Wongrapee:** Project administration; resources; supervision. **Patama Suttha:** Project administration; resources; supervision. **Phunlerd Piyaraj:** Project administration; resources; supervision. **Chaniya Leepiyasakulchai:** Investigation; methodology; resources; supervision. **Chuleeekorn Tanathitikorn:** Project administration; resources; supervision. **Pornsak Yoocharoen:** Project administration; resources; supervision. **Anthony Jones:** Project administration; resources; supervision. **Duangrat Mongkolsirichaikul:** Investigation; resources; validation. **Matthew Westercamp:** Methodology; resources. **Eduardo Azziz‐Baumgartner:** Conceptualization; methodology; resources. **Joshua Mott:** Conceptualization; funding acquisition; resources. **Suthat Chottanapund:** Methodology; project administration; resources; supervision.

### PEER REVIEW

The peer review history for this article is available at https://publons.com/publon/10.1111/irv.12975.

## DISCLAIMER

The findings and conclusions in this report are those of the authors and do not necessarily represent the official position of the U.S. Centers for Disease Control and Prevention or the U.S. Government. Material has been reviewed by the Walter Reed Army Institute of Research. There is no objection to its presentation and/or publication. The opinions or assertions contained herein are the private views of the authors, and are not to be construed as official, or as reflecting true views of the Department of the Army or the Department of Defense. The investigators have adhered to the policies for protection of human subjects as prescribed in AR 70‐25.

## Supporting information


**Table S1.** Reported underlying medical conditions at enrollment among 144 study participants who reported at least one condition and enrolled into a healthcare provider cohort, Bangkok, Thailand (2021)

## Data Availability

The datasets used and/or analyzed during the current study are available from the corresponding author on reasonable request.
