# Supplementary material for: Anti‐SARS‐CoV‐2 IgG antibody levels among Thai healthcare providers receiving homologous and heterologous COVID‐19 vaccination regimens
Source: Influenza Other Respir Viruses. 2022 Feb 24;16(4):662–72. doi: 10.1111/irv.12975 (PMC9111827; doi:10.1111/irv.12975)
Supplement: Supplementary file 1 — Table S1. Reported underlying medical conditions at enrollment among 144 study participants who reported at least one condition and enrolled into a healthcare provider cohort, Bangkok, Thailand (2021) [file IRV-16-662-s001.docx]

**Supplementary table 1. Reported underlying medical conditions at enrollment among 144 study participants who reported at least one condition and enrolled into a healthcare provider cohort, Bangkok, Thailand (2021)**

| **Condition** | **Number (%)** |
| --- | --- |
| Metabolic diseases (including diabetes) | 79 (54.9) |
| Hypertension | 50 (34.7) |
| Bone diseases e.g., osteoporosis, arthritis | 10 (6.9) |
| Heart and circulatory diseases (excluding hypertension) | 8 (5.6) |
| Asthma | 7 (4.9) |
| Liver diseases | 7 (4.9) |
| Hemoglobinopathy including thalassemia | 4 (2.8) |
| Neurologic/Neuromuscular disorder (including muscular dystrophy, cerebral palsy) | 4 (2.8) |
| Others^†^ | 9 (6.2) |

^†^Vertigo, depression, vitiligo, glaucoma, gastroesophageal reflux disease, and ovarian endometrioma
